# Beneficial effects of treatment with sensory isolation in flotation-tank as a preventive health-care intervention – a randomized controlled pilot trial

**DOI:** 10.1186/1472-6882-14-417

**Published:** 2014-10-25

**Authors:** Anette Kjellgren, Jessica Westman

**Affiliations:** Human Performance Laboratory, Karlstad University, Karlstad, Sweden; Department of Psychology, Karlstad University, SE 651 88 Karlstad, Sweden

**Keywords:** Flotation tank, Flotation, Relaxation response, Stress, Health, Sick leave

## Abstract

**Background:**

Sensory isolation in a flotation tank is a method known for inducing deep relaxation and subsequent positive health effects for patients suffering from e.g. stress or muscle tensions pains. Very few studies have investigated this method as a preventive health-care intervention. The purpose of this study was to evaluate the effects in healthy participants after receiving a series of flotation tank treatment.

**Methods:**

Sixty-five participants (14 men and 51 women) who were all part of a cooperative-health project initiated by their individual companies, were randomized to either a wait-list control group or a flotation tank treatment group where they participated in a seven weeks flotation program with a total of twelve flotation sessions. Questionnaires measuring psychological and physiological variables such as stress and energy, depression and anxiety, optimism, pain, stress, sleep quality, mindfulness, and degree of altered states of consciousness were used. Data were analysed by two-way mixed MANOVA and repeated measures ANOVA.

**Results:**

Stress, depression, anxiety, and worst pain were significantly decreased whereas optimism and sleep quality significantly increased for the flotation-REST group. No significant results for the control group were seen. There was also a significant correlation between mindfulness in daily life and degree of altered states of consciousness during the relaxation in the flotation tank.

**Conclusions:**

It was concluded that flotation-REST has beneficial effects on relatively healthy participants.

**Trial registration:**

Australian New Zealand Clinical Trials Registry: ACTRN12613000483752.

## Background

Stress-related ill-health such as depression, anxiety, and insomnia are all common reasons for sick-leave absence from work [1, 2] and stress-related illnesses have been studied in workplace settings [3–5]. Stress-related symptoms found amongst many employees include fatigue, burnout syndrome and gastric complaints [6] Stress, which is the most common reason for sick-leave absence [2, 3], may increase the number of days in sick-leave [4, 5], reduce productivity [5], and increase the risk of psychiatric disorders [7].

Occupationally induced fatigue play a major role in work-related psychological illnesses and research show that insufficient opportunities to recover from work fatigue contribute to stress-related illnesses [4]. One way of recovering from and/or preventing stress-related illnesses could be through health promoting programs at work places [4, 5] where relaxation techniques are used as a method. Health-care programs, including methods of stress-reduction such as relaxation, may be of such importance it will affect the number of sick-leave days [5]. Finding an effective method that can be implemented at work places might help individuals to find tools to prevent ill health and psychological negative factors. Evidence shows a positive effect on sick leave when health-care programs are implemented at work places [5]. Unfortunately, research regarding stress-related illnesses is mostly focused on those individuals already suffering, when preventive methods might be the most sufficient way of actually decreasing potential sick-leave absence by increasing general health [8].

Sick leave from work creates a heavy economic burden on the individual as well as the company, and it puts a mental strain on the person affected, colleagues and so on. There is a need to decrease the number of sick leave, especially those of stress-related character since they account for a large part of the sick-leave cost [9].

The physiological process of relieving the stress reaction is known as the ‘relaxation response’ (RR) [10, 11]. The relaxation response is attainable at a state of deep relaxation, and is the opposite of the “fight-or-flight” response. Why RR is such an effective remedy to stress symptoms is due to the direct link with parasympathetic nervous system activity where it lowers the heart rate and blood pressure as well as reducing respiratory frequency [11–13]. In order to successfully elicit the RR response in a stressful situation, it is critical that sensory input and bodily movements are reduced [10]. Unfortunately, many people find it difficult to engage in relaxation exercises that will generate RR [14]. One effective method to elicit RR is through relaxation in a flotation tank. During flotation-REST (Restricted Environmental Stimulation Technique) an individual lay in a horizontal floating posture immersed in highly concentrated salt water (magnesium sulphate) in a flotation tank. All incoming stimuli are reduced to a minimum during this period (usually 45 minutes), i.e. sound and light, and the water is heated to skin temperature. Flotation-REST has been scientifically investigated and is today considered a well-developed and scientifically proved method used to reduce stress, depression, anxiety, and to increase optimism and sleep quality [15–17]. Significant pain reduction has also been reported after using flotation-REST technique [18, 19]. A meta-analysis by Dierendonck and Nijenhuis [20] concluded that flotation-REST has positive effects on physiology (e.g., lower levels of cortisol, lower blood pressure and well-being). Flotation-REST appears to be an effective treatment method and more effective than other relaxation techniques included in their study (i.e. muscle relaxation, biofeedback, and meditation [20].

Earlier research regarding flotation-REST mainly involves individuals with a diverse array of ailment, and so there is a need to further investigate this method when used by working professionals in order to find out if it is a suitable method for preventing stress and stress-related problems in healthy participants.

The purpose of this study was to evaluate psychological effects in a series of treatments of flotation-REST in healthy participants. Based on previous studies, we hypothesized that beneficial effect on levels of pain, depression, anxiety, stress, energy, optimism and sleep quality would occur. Would it have beneficial effects on their health as proven in previous studies [20].

## Methods

### Participants

Sixty-five participants (14 men, 51 women) from three different companies with a mean age of 47.95 years (*SD* = 9.47) were included in the study. The participants were randomly assigned to a flotation-REST group (37 persons) or a wait-list control group (28 persons). Their mean number of monthly consumption of cigarettes was 47.23 (*SD* = 139.75), mean monthly consumption of alcohol was 172.90 millilitres (*SD* = 203.33) and mean number of used snuffboxes was 0.77 (*SD* = 2.87) every month. There were no significant differences regarding gender, age, cigarettes, snuff or alcohol between the two groups (independent samples t-tests, *ps* >0.256). The participants were all part of a cooperative-health project initiated by their individual companies. There was a wide range of occupational groups varying from managers, employers and employees all in the retail industry. The three companies had no part in any of the practical details regarding data collection, interpretation of results, study design etc.

### Design

A two-way split-plot design was carried out, where *Time* with assessments before and after the treatment sessions constituted the within-subject factor. *Group* (control group and flotation-REST group) constituted the between-subject factors. The participants were treated with flotation-REST during a 7-week period with a total of 12 flotation-REST sessions (45 min each). Several measurements (depression, anxiety, stress, energy, sleep quality, pain, optimism) were assessed before and after the treatment period. For the control group the same measurements were assessed before and after a 7-week period. After the period the control group also received flotation-REST treatment.

This study is registered as a clinical trial in Australian New Zealand Clinical Trials Registry (ANZCTR), as ACTRN12613000483752, and ethical approved by Karlstad University Ethical Review Board, dnr C2013/88.

### Instruments

#### SE - Stress and energy

This is a self-estimation instrument regarding an individual’s energy and stress experiences [21]. It is based on two subscales that indicate the mood levels regarding: 'experienced stress' and ‘experienced energy’. The subjects are to answer certain statements that are placed on a six-grade scales, from 0 = not at all, to 5 = very much. Examples of the statements are “How much do you feel relaxed”, “How much do you feel tense”. The SE-scale is based on an early checklist, the Mood Adjective Check-List [22] and has been further modified and translated into Swedish [23]. The instrument has been validated in a number of studies i.e. [21, 23]. Cronbach's alpha was 0.84 for the stress subscale, and 0.74 for the energy subscale. The score range is from 0 – 5 for each subscale.

#### HADS - Hospital anxiety depression scale

The HADS is a rating scale regarding degree of anxiety and depression. It was constructed for use with physically ill people [24], and has been validated and reliability tested [25]. The HAD scale consists of fourteen statements with four response alternatives ranging from 0 to 3. Examples of the questions are ““Do you feel tense or wound up?”, “Do you take as much interest in things as you used to?” There are seven statements concerning anxiety and seven for depression, wherein values under 6 are considered normal, those between 6 and 1 are borderline, and all values over 8 points are indicative of a probable depression or anxiety diagnosis [26]. The score range is from 0 – 21 for each subscale. Cronbach's alpha was 0.74 for the depression subscale, and 0.82 for the anxiety subscale.

#### LOT - Life orientation test

The test [27] consists of eight items plus four filler items. The task of each participant is to decide whether or not one agrees with each of the items described. The scale ranges from 0 – 4 where 0 indicates “strongly disagree” and 4 indicates “strongly agree”; the total score range is 0 – 32. The test measures dispositional optimism, defined in terms of generalized outcome expectancies. Parallel Test Reliability is reported to 0.76 and Internal Consistency to 0.76 [27] and Test-Retest reliability to 0.75 [28]. LOT is also regarded as having an adequate level of convergent and discriminant validity [26] as demonstrated by correlation statistics and by using LISREL VI (*r* = 0.64).

#### SQ - Sleep quality

This instrument consists of 11 questions that elucidate the sleeping habits [29]. Responses to 9 questions are placed on a 0–4 scale, response to one question is placed on a 0–5 scale, and response to one further question is placed on a 0–8 scale. Examples of the questions are “How often do you feel tired on week days?”, “How often do you feel you did not get enough sleep?”, “How do you feel you usually sleep?”. The psychometric properties were examined by comparing healthy and sick people, and using Cronbach’s alpha (alpha = 0.88) [28]. Score range is 0 – 45, and Cronbach’s alpha was 0.64.

#### MAAS

Mindful Attention Awareness Scale is designed to assess the main characteristic of dispositional mindfulness [30]. It measures whether a person is open or receptive to what is happening in the present and has a mindful state over time through a 15-item scale [30]. The instrument consists of 15 items, all of which indicate a lack of mindfulness. The items are rated on a 6-point Likert scale ranging from 1 (almost always) to 6 (almost never). If the scores are rated high – it indicates more mindfulness of the subject (the total score can range from 15 to 90) [31]. Cronbach’s alpha was 0.80.

#### Normal pain and worst pain

Visual Analog Scale (VAS). These scales were used for measuring normal pain and experienced worst pain. They consisted of a 100 mm horizontal line with the anchors “no pain” on the left extreme and “excruciating pain” on the right extreme. VAS is considered the “gold standard” for assessment of clinical pain, and changes in VAS score are regarded as significant evidence of individual response to treatment, placebo, or experimental manipulation [32]. The accuracy and precision have been examined for both clinical and experimental pain, and found adequate [33].

#### EDN - Experienced deviation from normal state

This is an instrument that is constructed to be used in flotation-REST experiments based on the APZ-questionnaire and OAVAV [34] so that an assessment of altered states of consciousness and the relaxation response can be made. The EDN consists of 29 questions and these statements should each be responded to on a VAS-scale 0–100 mm (where 0 means “No, not more than usually” and 100 means “Yes, much more than usually”). Points obtained from these statements, are averaged so that an “index of experience” (0 – 100) can be measured [16]. Through this, the total experience of deviation from normal states can be obtained. Examples of statements are “It seemed to me that my environment and I were one” and “I experienced past, present and future as an oneness”.

In earlier studies e.g. [11, 35] the EDN scale has been used where Cronbach’s alpha measured between 0.91 – 0.97, which suggests very high reliability for this scale; in the present study Cronbach’s alpha was 0.93. The average EDN-value following a person’s first encounter with flotation-REST is approximately around 30. Resting on a bed in a dark quiet room generates approximately 15 points [36].

#### Flotation tank

Outer measurements are 2600 × 1650 × 1330 mm. In total it contains 600 litres of water and 350 kg’s of magnesium sulphate salt. To maintain a correct water temperature (approximately outer skin temperature, 35°C), heating foil is placed in the lower section of the floating tank, regulated by a thermostat and heated in regular intervals. The door is outwardly opened. The inside of the lower section had ribbed bottom to prevent slipperiness. Between flotation sessions, water is filtered and sterilized with UV-light along with weekly addition of hydrogen peroxide where oxidization occurs. The sterilization of water between flotation sessions takes approximately fifteen minutes. Filters are regularly changed and the inside of the flotation tank is cleaned twice a week in accordance to public health board recommendations.

### Procedure

An agreement was made between the owner of a flotation centre and three managers from three different companies about a health-care program involving flotation-tank treatments. All three companies advertised through intranet correspondence, their company magazines, and through emails, offering all employees to participate in the health-care program. All interested participants were invited to an information meeting at the flotation centre located in Deje in the county of Varmland, Sweden, where they were further informed about the program. Information was given that the flotation-rest program would enable them to participate in a scientific study concerning the effects of this treatment program. Further information was given regarding that the participation was voluntary, confidential, that they would be randomly selected to either experimental group or waiting list, and that they could terminate their participation at any given point. All participants made a verbal informed consent about participating. They were also informed it was possible to participate in the health-care program without participation in the present study. All participants filled out a questionnaire regarding basic facts (age, gender, alcohol consumption, nicotine use) as well as SE, HADS, LOT, VAS and SQ questionnaires. Data was collected by the manager of the flotation center.

After the introduction and data collection, the enrolled individuals were randomly assigned to either the control group or to the flotation-REST group (a total of 12 flotation sessions). Those individuals that had been randomized to the wait-list control group got the opportunity to later participate in flotation-treatments after the present study was terminated.

All participants were guided through the flotation centre. Participants in the flotation-REST group booked the 12 awaiting flotation sessions (around two per week for a period of seven weeks) where each session was of 45 minutes duration and 30 minutes to shower and relax afterwards. After the third flotation session a supplementary EDN questionnaire was filled out by the experiment group. After the last flotation-REST session, the questionnaires (i.e., SE, HADS, LOT, VAS, EDN and SQ) were once again answered by the flotation-REST group along with a complementary MAAS questionnaire. All questionnaires were filled out in privacy in a separate room (no staff there) and the participant put their questionnaires in a closed box, that were later transported un-opened to the researchers.

The control group was scheduled to return 7 weeks later for filling in the scales SE, HADS, LOT, PAI, and SQ again. All persons were treated in accordance with the Declaration of Helsinki, Ethical Principles for Medical Research Involving Human Subjects. Time estimated between first announcement of health project and completion was about 6 months. The participants suffered from no sick absence and were hence referred to as being relatively healthy.

## Results

### Stress-related psychological variables

A two-way mixed Pillais’ MANOVA was carried out with Time (before, after) as the within-subjects factor and Group (flotation, control) as between-subjects factor. Dependent variables were the psychological variables which in earlier studies e. g. [29] have been proven to strongly intertwine, i. e., stress (SE), energy (SE), anxiety (HADS), depression (HADS), dispositional optimism (LOT), and sleep quality (SQ). The analysis showed significant effects for Time (*p* <0.001, *Eta*^*2*^ = 0.48) and for Time × Group interaction (*p* = 0.008, *Eta*^*2*^ = 0.26). There was no significant effect for Group (*p* = 0.248, *Eta*^*2*^ = 0.13). The results from the univariate F-tests are given below. For means and standard deviations, see Table 1.

**Table 1:** **Means (and Standard deviations) for the psychological variables before and after the treatment period**

***Variable***	***Before***	***After***	***Difference***
SE Stress control	1.84 (1.15)	1.89 (1.04)	+ 0.05 n.s
SE Stress floating	1.86 (1.07)	0.95 (0.84	- 0.91 **
SE Energy control	3.44 (0.70)	2.63 (0.96)	- 0.81 n.s
SE Energy floating	3.14 (0.66)	2.46 (1.02)	- 0.74 n.s
HADS Anxiety control	7.03 (3.46)	6.96 (3.52)	- 0.07 n.s
HADS Anxiety floating	7.92 (4.61)	4.28 (3.61)	- 3.64 **
HADS Depression control	4.00 (3.41)	4.30 (2.58)	+ 0.30 n.s
HADS Depression floating	4.42 (3.47)	2.25 (2.53)	- 2.17 **
LOT optimism control	20.96 (5.05)	20.93 (5.76)	- 0.03 n.s
LOT optimism floating	19.81 (5.29)	23.28 (4.26)	+ 3.47 **
SQ Sleep Quality control	25.22 (9.98)	25.33 (8.87)	+ 0.11 n.s
SQ Sleep Quality floating	23.72 (7.55)	29.69 (8.44)	+ 5.97 **

** = significant effect, p < 0.001; n.s = non significant effect.

### Stress

The analyses yielded a significant difference for Time [*F* (1, 61) = 6.44, *p* = 0.014], and a descriptive analysis showed that stress was reduced from 1.85 (*SD* = 1.10) to 1.35 (*SD* = 1.04) after the treatment period. There was also a significant interaction Time x Group [*F* (1, 61) = 8.23, *p* = 0.006], and further analysis (pair-samples t-tests, 5% level) showed that the stress was reduced for the flotation group (*t*_(36)_ = 4.42, *p* <0.001) but not for the control group (*t*_(27)_ = 0.169, *p* = 0.867).

### Energy

The analyses yielded a significant effect for Time [F (1, 61) = 20.70, p <0.001], where the energy was lowered from 3.27 (*SD* = 0.69) before the treatment, to 2.54 (SD = 0.99) after the treatment. There was no significant interaction Time × Group (*p* = 0.707) indicating both groups lowered their energy-level in a similar way.

### Anxiety

The analyses yielded a significant difference for Time [*F* (1, 61) = 19.42, *p* <0.001], and a descriptive analysis showed that the anxiety was reduced from 7.54 (*SD* = 4.15) to 5.43 (*SD* = 3.3.79) after the treatment period. There was also a significant interaction Time × Group [*F* (1, 61) = 17.90, *p* <0.001], and further analysis (paired-samples t-tests, 5% level) showed that anxiety was reduced for the flotation group (*t*_(36)_ = 5.67, *p* <0.001) but not for the control group (*t*_(27)_ = 0.231, *p* = 0.819).

### Depression

The analyses yielded a significant difference for Time [*F* (1, 61) = 7.68, *p* <0.001], and descriptive analysis showed that depression diminished from 4.24 (*SD* = 3.42) to 3.13 (*SD* = 2.73) after the treatment period. There was also a significant interaction Time × Group [*F* (1, 61) = 13.32, *p* = 0.001], and further analysis (paired-samples t-tests, 5% level) showed that depression was reduced for the flotation group (*t*_(36)_ = 4.74, *p* <0.001) but not for the control group (*t*_(27)_ = 0.508, *p* = 0.616).

### Optimism

The analyses yielded a significant difference for Time [*F* (1, 61) = 9.83, *p* = 0.003], and a descriptive analysis indicated that optimism increased from 20.30 (*SD* = 5.18) to 22.27 (*SD* = 5.06) after the treatment period. There was also a significant interaction Time × Group [*F* (1, 61) = 10.26, *p* = 0.002], and further analysis (paired-samples t-tests, 5% level) showed that optimism was increased for the flotation group (*t*_*(*36)_ = 4.36, *p* <0.001) but not for the control group (*t*_(27)_ = 0.06, *p* = 0.953).

### Sleep quality

The analyses yielded a significant difference for Time [*F* (1, 61) = 10.11, *p* = 0.002], and a descriptive analysis indicated that sleep quality increased from 24.37 (*SD* = 8.64) to 27.83 (*SD* = 8.83) after the treatment period. There was also a significant interaction Time x Group [*F* (1, 61) = 9.38, *p* = 0.003], and further analysis (paired-samples t-tests, 5% level) showed that sleep quality was increased for the flotation group (*t*_(36)_ = 3.95, *p* <0.001) but not for the control group (*t*_(27)_ = 0.323 , *p* = 0.749).

Correlation analyses (Pearsons’) between the variables Stress and Sleep quality were also performed, and there was a significant negative correlation both before intervention (*r* = -0.585, *p* <0.001) and after intervention (*r* = -0.507, *p* <0.001).

### Pain variables

Two repeated measures ANOVA were carried out with Time (before, after) as the within-subjects factor and Group (flotation, control) as between-subjects factor. Dependent variables were worst pain and normal pain. See Table 2.

**Table 2:** **Means (and Standard deviations) for the pain variables before and after the treatment period**

***Variable***	***Control group***	***Flotation-REST group***
VAS Worst pain before	64.76 (25.14)	64.29 (28.12)
VAS worst pain after	55.15 (28.97)	39.70 (32.11)
VAS normal pain before	30.28 (21.92)	27.32 (20.12)
VAS normal pain after	25.68 (17.15)	15.00 (17.17)

### Pain (worst pain)

The analyses yielded a significant difference for Time [*F* (1, 58) = 28.672, *p* <0.001] and a descriptive analysis showed that worst pain decreased from 64.29 (*SD* = 28.13) to 39.70 (*SD* = 32.11) after treatment period. There was also a significant interaction between Time × Group [*F* (1, 58) = 5.494, *p* = 0.023], where the pain decreased from 64.29 (S*D* = 28.12) to 39.70 (*SD* = 32.11) for the flotation group; for the control group there were no significant differences (pain before = 64.76, pain after = 55.15).

### Pain (normal pain)

There were no significant differences or interactions for normal pain (*p* >0.05).

### Mindfulness and Altered states of consciousness

**Figure 1:**
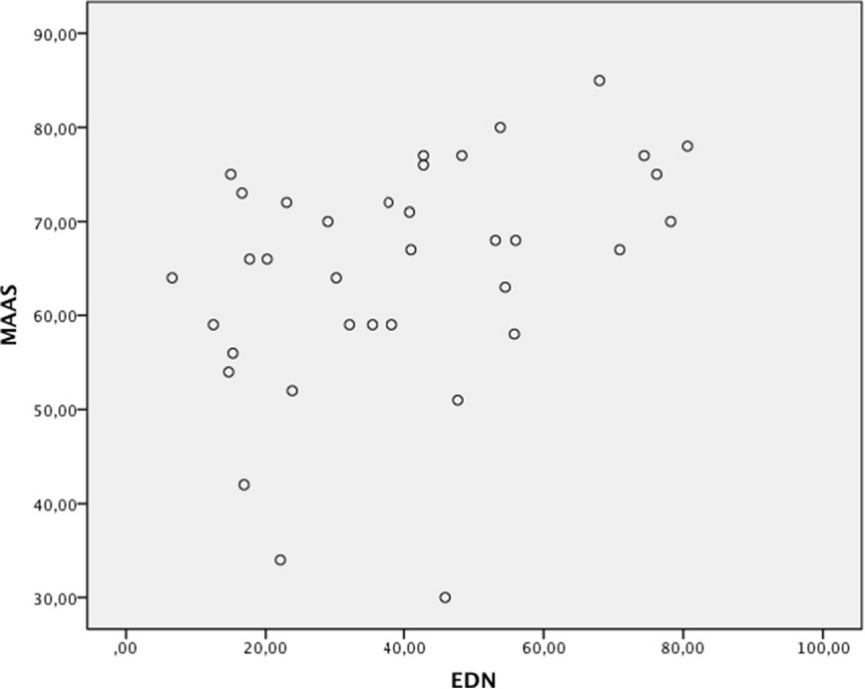
**Scatterplot for MAAS vs EDN.**

There was a significant correlation between Mindfulness (as measured with MAAS) and degree of Altered states of consciousness (as measured with EDN), *r* = 0.391, *p* = 0.018. For a scatter plot for MAAS vs. EDN, see Figure 1.

In order to further evaluate this correlation the variable EDN was divided into three parts (low, medium, high) thereby creating the independent variable EDN-group. Cut-off points for these levels were 33.3% and 66.7%. Analysis with one-way ANOVA with EDN-group (low, medium, high) as the independent variable, and MAAS as dependent variable yielded a significant difference between the groups [*F* (2,35) = 4.033 *p* = 0.027, *eta*^*2*^ = 0.39]. A post–hoc analysis (Bonferroni) showed there was a significant difference between “low” and “high” (*p* = 0.029) but not with comparison to the medium condition (*ps* = 0.163); the persons obtaining a higher degree of altered states of consciousness in the tank also experience more mindfulness in their daily life.

## Discussion

The purpose of this study was to evaluate psychological effects in a series of treatments of flotation-REST in healthy participants (i.e. not being on sick-leave absence). The main findings were significant decreased experienced stress, worst pain, anxiety, and depression - as well as significant increased sleep quality and optimism for the flotation-REST group compared to the control group. In addition, it was found that the dimensions mindfulness and altered states of consciousness, at least to some extent, seemed to be overlapping constructs.

Flotation-REST treatment could be a valuable tool for increased over-all wellbeing. The effect-size for the performed general MANOVA-analysis showed a large effect, and therefor probably indicating a beneficial effect of real importance. General sleep quality significantly increased after flotation-REST treatment. There was also a significant decrease in experienced stress for the flotation-REST group, which may be a contributing factor to the increased sleep-quality. In order to successfully fall and stay asleep a relaxed and stress-free state is important. There was also a significant decrease in experienced stress for the flotation-REST group, which was directly correlated with increased sleep-quality.

By having experienced deep relaxation through flotation-REST, it could help a person to find a similar state of relaxation in daily life. Sleep deprivation is known to have negative effects on certain cognitive functions [37], being a trigger for depression and other psychological diseases, as well as reducing the immune defence [38, 39]. Sleep deprivation has also shown to be a trigger of hypertension, heart disease, and diabetes [40, 41], which are all serious health issues. Lack of sleep is also a contributor to sick-leave absence from work [41]. Being able to increase sleep quality and quantity is advantageous on an individual basis and might prevent possible sick leave. There was also a significant lowered energy level over time (for both groups); we have no explanation for this.

Flotation-REST also decreased the experienced “worst pain” intensity. Decreased pain in participants using flotation-REST has been extensively described in earlier studies e.g. [11, 17, 42]. Participants in this present study were not pain patients, but did however experience some pain in different parts of the body that apparently diminished significantly. It can be assumed that many of the experienced pain were due to muscle tensions, and that the induced relaxation and stress reduction in the tank may be a possible explanation for reducing these pains. There were no decrease in the experienced “normal pain”; maybe such low-intensity pains are more due to other reasons than muscle tensions, and therefor not affected by the deep relaxation in the tank.

Degree of anxiety and depression did also significantly decrease for the flotation-REST group during the treatment weeks. Depression and anxiety are strong contributors to sick-leave and serious health problems such as high blood pressure [40]. In addition, it was concluded that optimism significantly increased for the flotation group, which is a further indication of general wellbeing and psychological health. Flotation-REST treatments seem to enhance wellbeing and could certainly have great value and importance as a health-care method.

Another new finding was that the dimensions mindfulness (as measured with MAAS) and altered states of consciousness (as measured with EDN) seemed to be partly overlapping constructs. Those individuals who reported higher levels of altered states of consciousness (ASC) during flotation-REST also scored high on MAAS (and thus were open and receptive to here-and-now and had a mindful state in daily life). To our best knowledge, this is the first time the correlation between ASC and mindfulness has been investigated. We have in earlier studies [16, 17, 19] argued that degree of ASC in the flotation tank is a measure of the relaxation response and a person’s ability to feel secure. A recent review [43] of empirical studies concludes that mindfulness is closely associated with psychological well-being. Several studies (e.g. [17, 20] have earlier confirmed the beneficial effects of flotation-REST, and it has been suggested that the deep relaxation and stress reduction is the main contributing factors. From the present study we would also like to suggest that flotation tank treatment facilitates the psychological process of increased mindfulness, but further studies are needed for confirming if such causality exists.

Many of the participants wrote comments, without being asked to do so, on their individual questionnaires. They continuously emphasized how their pain - which many of them had been having for many years - was practically gone after the 12-session flotation program. They mentioned further how they felt relaxed, slept better, and were over-all happier and healthier. Many of the participants had prior to the floating sessions been using a range of different methods to reduce pain, stress and other individual health issues. Medicines, yoga, massage, and physiotherapy were some of the treatments mentioned, and never had they so successfully been relieved from pain, tension, stress etc.

## Conclusions

The results of the present study indicate that flotation-REST may reduce contributing factors to potential stress-related illness as well as increase certain psychological factors in healthy participants. Stress, anxiety, depression, and worst pain decreased and sleep quality and optimism increased in the flotation-REST group compared to the wait-list control group. This technique might increase general health and thus help prevent future sick leave. On an individual basis, sick leave puts a negative strain on the affected. Not only financially but it also tends to generate further negative effects such as lack of career possibilities, confidence, and a decrease of social interactions. Whether or not flotation-REST has positive effects on reduction of sick leave need to be evaluated in further research.
